# Patient and healthcare professionals’ perceptions of educational tools to reduce urine culture contamination in outpatient clinics: a qualitative study

**DOI:** 10.1017/ash.2026.10317

**Published:** 2026-03-09

**Authors:** Ashley Collazo, Trenton M. Haltom, Johanan Luna Rodriguez, Kiara Olmeda, Azalia Mancera, Fabrizia Faustinella, Michael K. Paasche-Orlow, Zach Landis-Lewis, Roger Zoorob, Barbara W. Trautner, Larissa Grigoryan

**Affiliations:** 1 Baylor College of Medicine Department of Family and Community Medicine, USA; 2 Baylor College of Medicine Department of Medicine, USA; 3 Washington University in St Louis Division of Infectious Diseases, USA; 4 Tufts University School of Medicine, USA; 5 University of Michigan Medical School, USA

## Abstract

**Objective:**

Iteratively develop educational tools (instructional video and flyer) to improve midstream clean catch (MSCC) urine sample collection using patient and healthcare professionals’ input.

**Design:**

Multi-method qualitative study.

**Setting:**

Outpatient clinics in Houston, Texas, United States.

**Participants:**

Adult patients recruited from public and private clinics (*n* = 12). Healthcare professionals (HCP; nurses and medical assistants) (*n* = 12) providing care at participating clinics.

**Methods:**

Twelve patient interviews and three focus groups with HCPs (May 2024–November 2024). Interviews discussed patient experiences using the educational tools to guide urine specimen collection. Focus groups elicited HCPs’ perspectives on the comprehensibility and utility of the tools in their respective clinics. We identified themes using directed content analysis.

**Results:**

We garnered insight into knowledge gaps and barriers for completing the MSCC process. MSCC instructions in existing educational tools were poorly understood by patients, especially among those with limited understanding of urogenital anatomy. Patient barriers to MSCC collection included physical difficulties due to poor urine stream control, mobility issues, and obesity. Patients and HCPs reported that our tools addressed patient gaps in understanding of MSCC instructions. Patients and HCPs also suggested that we accompany our tools with assistive devices and dedicated surfaces in the clinic bathrooms, to better meet patients’ needs in urine specimen collection.

**Conclusions:**

Initial feedback was promising that our educational tools would improve the MSCC collection process for patients. In next steps, we will conduct feasibility pilot testing followed by a randomized controlled trial to test the effectiveness of reducing urine culture contamination.

## Introduction

Urinary tract infections (UTI) result in more than 10 million ambulatory visits per year and are the third-leading cause for outpatient antibiotic prescriptions.^1^ With the rise of resistant urinary pathogens, urine cultures are essential for guiding providers’ antibiotic choices. Urine contamination during collection, however, is common; some laboratories report a urine culture contamination rate of more than 40%.^2–4^ Contamination leads to incorrect diagnoses, inappropriate treatment, and high costs.^2^ Indeed, two studies in United States (US) outpatient safety-net clinics found more than half of urine cultures were contaminated (55%) and 1-in-5 patients were unnecessarily treated with antibiotics.^5,6^

Patients rarely perform the steps for midstream clean catch (MSCC) urine collection correctly in part due to lack of appropriate instruction.^7,8^ A study of US emergency departments found correct MSCC technique in only 6% of cases, with 57% of patients receiving no urine collection instructions.^8^ A survey of English- and Spanish-speaking female patients found only 61% reported receiving any instructions.^7^ The two important steps in preventing contamination—instructions on parting the labia and collecting a midstream sample^9^—were given only to 36% and 16% of patients, respectively.^7^

Interventions to improve patient instruction around the MSCC urine collection have mixed results.^10–13^ Some interventions failed to reduce urine contamination,^10,11^ while others successfully decreased contamination.^12,13^ However, these interventions were developed without healthcare professional (HCP) or patient input and did not include visual aids such as animated videos. With HCP and patient input, we developed patient educational tools for MSCC urine collection in English and Spanish that included animated instructional videos and flyers with pictorial instructions. Through qualitative analyses of interviews and focus groups, we aimed to understand patients’ experiences providing MSCC urine samples and use this information to iteratively develop tools to improve MSCC sample collection.

## Methods

### Design

We took a multi-method qualitative approach (interviews and focus groups) to elicit patients’ and HCPs’ thoughts on the tool’s usefulness in completing the MSCC process.

### Sample selection and recruitment

We used purposive sampling to recruit patients and HCPs from public and private healthcare systems. Our patient recruitment strategy involved a presentation to the Harris Health System (HHS) Patient Council At Large meeting in Houston, TX. This meeting enables Harris County patients to provide feedback on studies conducted in the safety-net clinics of HHS.^14^ Patients at this meeting were invited to be a part of our study and provided contact information if interested. Additional patients were recruited through private family medicine clinic staff referral. We recruited English- and Spanish-speaking patients to ensure representation of their perspectives and experiences in the development and refinement of the tools. All patients were screened for eligibility (≥18 years of age and Harris Country residents who spoke English or Spanish). Interviews occurred between May and June 2024.

We also completed virtual focus groups with nurses and medical assistants employed by the participating clinics, which we refer to as healthcare professionals. HCP recruitment was done by referral wherein clinic directors of public and private family medicine clinics recommended professionals for focus group participation. Focus groups were held between September and November 2024.

### Educational tools

**Figure 1.**
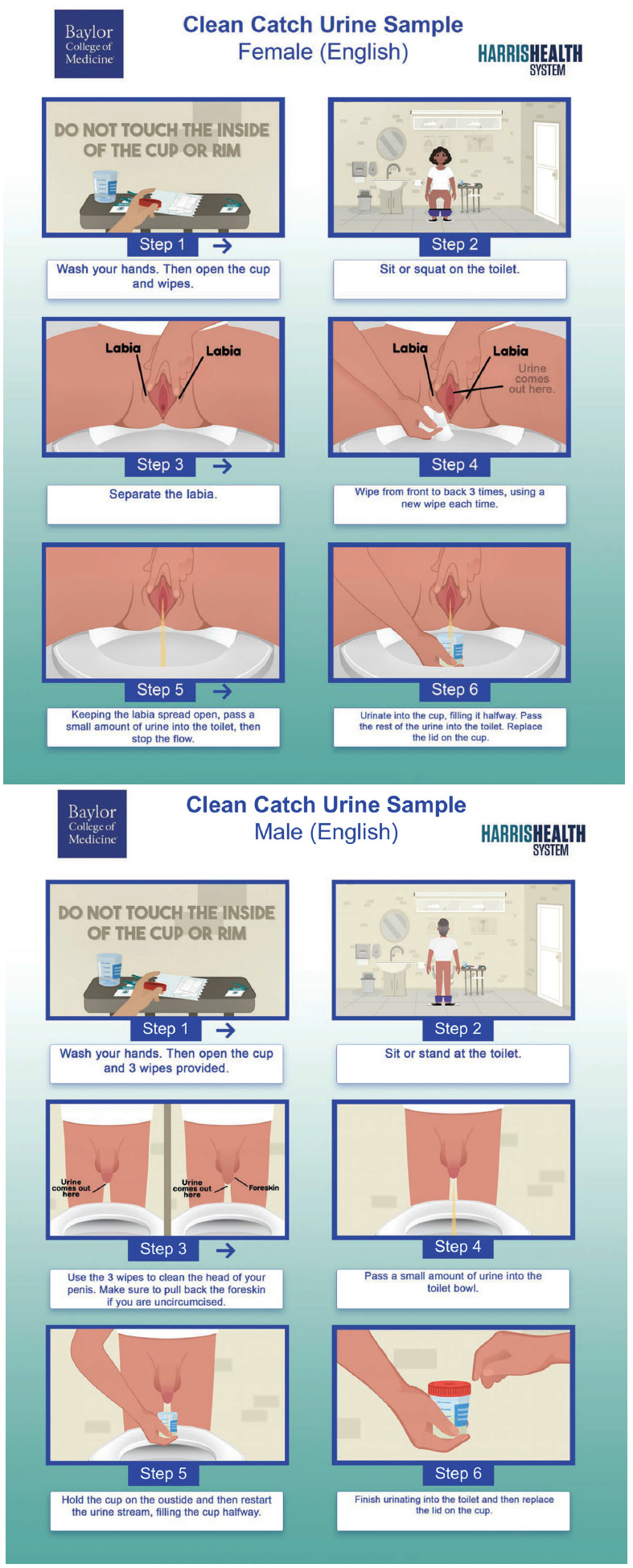
Instructional flyers detailing the six steps of the midstream clean catch process for both female and male patients.

Our educational tools included an animated instructional video and flyer with pictorial instructions. We developed the tools using previously published studies.^10–12^ The video and flyer were created by an external consulting media production agency.^15^ The video consisted of two minutes of animated instructions on the MSCC urine collection process with separate versions for men and women in English and Spanish (Supplemental Material 1). The flyer encompassed six steps with pictorial and written instructions for the MSCC process in English and Spanish for both sexes (Figure 1). Development of the tools was led by an author with experience in film writing, directing, and production (FF) and input from experts in health literacy (MPO) and Hispanic cultural and linguistic competency (MLA). Tools were then reviewed by the recruited patients and HCPs.

### Data collection

Interviews and focus groups followed guides (Supplemental Material 2) developed by study team members (LG, BWT, MLA, MPO, RC) with expertise in UTI-related antibiotic stewardship, qualitative research, implementation science, health literacy, and Hispanic cultural and linguistic competency.

Prior to the interview, patients were mailed a urine collection kit (a sterile collection container and three wipes) and the flyer. During the interviews patients discussed their experience using the urine collection kit and flyer. They also watched and provided input on the video. Interviews lasted 60 minutes. Patients were compensated US $75.

HCP focus groups met once virtually via Zoom with a qualitative analyst (RC). During the hour-long focus groups, the video and flyer were shown, and HCPs provided their thoughts on the comprehensibleness and utility of the educational tools. HCPs were compensated US $25.

All interviews and focus groups were recorded, manually transcribed, and verified for accuracy by two research team members. Data collection stopped once no new information was identified.^16^

### Data analysis

Data were analyzed using qualitative directed content analysis.^17,18^ Coders (AC, AM, KO, JLR) deductively developed a codebook upon review of the data and used iterative comparison to produce and name codes. Through continued review, we added codes inductively. Study team members discussed codes to ensure internal validity and to reach agreement on a final codebook. Transcripts were coded using ATLAS.ti.^19^ Discrepancies in coding were adjudicated between team members. All members of the research team contributed to defining and refining the themes developed from the data.

### Ethical considerations

This study was approved by the Institutional Review Board of Baylor College of Medicine (protocol #H-53559). Informed consent was obtained from all participants.

## Results

### Participant demographics

Interviewed patients were racially and ethnically diverse (n = 12, Table 1). Over half the interviewees were bilingual in English and Spanish (n = 7, 58%). There were equal numbers of male and female patients. HCPs included registered nurses (n = 4, 33%) and medical assistants (n = 8, 67%), all of whom were female.

**Table 1.** Demographic characteristics of patients and healthcare professionals

	Patients (n = 12)	Healthcare Professionals (n = 12)
	n (%)	n (%)
Sex		
Female	6 (50)	12 (100)
Male	6 (50)	0 (0)
Race/Ethnicity		
Non-Hispanic White	2 (17)	0 (0)
Non-Hispanic Black	3 (25)	4 (33)
Hispanic	7 (58)	7 (58)
Other	0 (0)	1 (8)
Clinics		
Public	6 (50)	---
Private	6 (50)	---
Role		
Nurse	---	4 (33)
Medical Assistant	---	8 (67)
Spoken Languages		
Bilingual (English-Spanish)	7 (58)	---

Patients participated in interviews and healthcare professionals participated in focus groups.

### Themes from patient interviews and healthcare professional focus groups

We developed three themes from interviews and focus groups: (1) limited patient knowledge of the MSCC; (2) tools demystify and ease the MSCC process; and (3) patient barriers to MSCC urine collection. Table 2 summarizes the content analysis from the reported themes. We attribute quotes from the patients with “PAT” followed by a participating number, sex, and race/ethnicity. We label quotes from healthcare professionals with “HCP” followed by participant number and role (ie, registered nurse or medical assistant). Themes focus on the collection process from the perspective of the patient or HCPs’ experiences with patients; therefore, the patient voice predominates across all three themes while HCP insight is limited to the barriers to MSCC collection.

**Table 2.** Summary of content analyses by theme

**Limited Patient Knowledge of the MSCC**	**n**
Previous experience providing urine sample	9
No prior instruction given	3
Previous instruction inconsistent with tools	5
Poor understanding of the MSCC	9
Unfamiliarity with medical terminology and anatomy	4
**Tools Demystify and Ease the MSCC Process**	
Tools were clear and understandable	12
Tools were comprehensive	4
Tools were useful or helpful	9
**Patient Barriers to MSCC Urine Collection**	
Physical difficulties preventing proper collection	9
Logistical barriers complicating collection	9
Recommendations to address barriers	15

n = patients or healthcare professionals.

MSCC = midstream clean catch.

### Limited patient knowledge of the MSCC: process, necessity, and anatomy

Patients’ descriptions of their previous experiences providing urine samples revealed their limited knowledge of how to complete a MSCC. Some patients reported never receiving proper instruction on the clean catch process wherein HCPs “just give you the cup…. They don’t give you the steps” (PAT08-Female-Hispanic). One patient felt clinic staff “just assumed that I knew” (PAT06-Male-Hispanic) the steps for collection.

Even when participants were given instructions, the instructions did not include all the steps listed in our educational tools. For example, patients stated they had “never done it like this” (PAT01-Male-White) or realized “Oh! Actually, I have not been doing urine samples correctly” (PAT06). Inconsistencies in MSCC instruction highlight incomplete standardization of information provided to patients across clinics.

Patients also did not understand the necessity of a clean catch. PAT06 was confused as to why it was “suggested to pass a bit of urine before… What’s the purpose for that?” This shows patients’ difficulty comprehending the importance of the midstream portion of the MSCC process. Another aspect which participants struggled to navigate was urogenital medical terminology and anatomy. This was often the case in female patients who expressed deficits in their anatomical education, “you have to remember that some of us were not educated as to our bodies growing up” (PAT12-Female-Hispanic). Another patient similarly commented how “when I was younger, I didn’t know I had three openings. I thought we had two” (PAT07-Female-Hispanic). Their knowledge was further limited to what was passed on from their mothers, “I never learned that. My mom didn’t teach me that” (PAT09-Female-White). This informal and insufficient education directly impacts the quality of urine collection.

### Tools demystify and ease the MSCC process

We learned that our educational tools demystified and eased the MSCC collection process for patients in three ways. First, patients found our video and flyer “very clear,” “simple to understand,” and “pretty straightforward.” PAT10 (Male-Hispanic) easily understood the written flyer instructions, saying how it contained “nothing very hard to read. I feel like someone that’s probably in middle school could read this and use basic instructions, anyone from any age.”

Second, in cases where patients could not read or when there was a language barrier, animated video and the pictorial instructions were useful. For example, PAT01 explained a common situation with his wife:

Some people don’t speak [English]. For example, my wife. I have to go with her to the provider, ask for an interpreter, [with the flyer] she can understand the pictures, [even though] she cannot read… the pictures would be enough.

The animated video and pictorial flyer instructions were also helpful in addressing the knowledge gaps around male and female anatomy found in patient interviews. Male patients appreciated the inclusion and labeling of the foreskin “in case somebody didn’t know what foreskin was, [the video] teaches you right away” (PAT03-Male-Hispanic). One female patient felt the video was particularly helpful in clarifying anatomical terminology. As she explained, “some might not understand this medical [word]. I thought these were the lips (referring to the labia), but the video really makes it quite clear” (PAT05-Female-Black). Ensuring patients identify these genital structures increases the possibility of correct MSCC collection.

Finally, patients reported our tools were comprehensive, providing all necessary steps for urine collection. PAT07 explained, “I like how it went step by step…it shows you exactly what needs to be done.” Having each step clearly laid out also increased patients’ ability to complete the steps correctly, illustrated by statements like “I feel more comfortable with the steps” (PAT08) and “everything provided helped to ease the process” (PAT06). Step-by-step instruction helped demystify and ease MSCC collection for patients.

### Patient barriers to MSCC urine collection

Patients and HCPs identified barriers to the MSCC process that our tools did not address. Among these barriers were physical difficulties that can prevent proper clean catch collection. Both male and female patients struggled at times to stop their urine stream for a midstream sample saying how “sometimes we cannot stop our pee” (PAT01) and “I’m not good at stopping my urine” (PAT07). PAT05 implied it could be difficult to coordinate movements to accurately collect a sample like using “the muscles [to] stop and start again” as well as making sure to “get back down there quick enough [with the cup].” Providers also cited mobility issues and obesity as barriers “because they are not able to reach that area” (HCP01-registered nurse) to adequately clean prior to collection or these patients struggle “balancing themselves [while] doing all the things” (HCP02-medical assistant) needed for accurate collection.

Physical barriers to urine collection were compounded by the fact that clinic bathrooms are often not configured to support sample collection. One female patient explains this interplay between the physical difficulties associated with collecting a MSCC and medical bathrooms:

I’m a heavy person and where my stomach is, when you bend down and try to do the steps like in the flyer, it’s really difficult to do…. When you go to some places like in the hospital, the toilets are very low… [or in the] clinic, the toilet is too close on one side, and you couldn’t really open your legs to do this step. (PAT02-Female-Black)

Both patients and providers mentioned limited space to place collection materials in bathrooms as a functional barrier complicating collection. For example, HCP03, a medical assistant, explained clinic bathrooms were “not made with counters for a patient to place items” which made it difficult to lay out collection materials. She explained that patients “pretty much only have the top of the toilet and paper holder to put stuff” which are not areas regularly cleaned after patients use the bathroom.

Among patients and HCPs, there were efforts to brainstorm solutions to these barriers. They recommended providing a place for collection materials and additional items to improve the collection process such as gloves, wider collection cups, and a urine collection hat.

Patients and providers also believed additional assistance should be offered to those that struggle with the process. Patients felt clinic staff should be available to answer questions or provide clarification. In PAT05’s words, “some people might need more instruction, side by side [with clinical staff].” HCPs recommended providers physically assist patients with impediments to proper clean catch collection. From HCP02’s experience as a medical assistant:

Sometimes there’s been a case where I’ve had to help a patient give us a clean catch…. I just held the cup there, when it was more than halfway, I removed the cup, and she went the rest of the way in the toilet…. She just had a stroke, so the left side of her body was not working.

Overall, patients and HCPs identified barriers to midstream clean catch collection and discussed possible solutions.

## Discussion

The purpose of our educational tools is to reduce urine culture contamination by providing clear, step-by-step instructions for a MSCC urine sample. Through patient interviews and healthcare professional focus groups, we found instructional inconsistencies in patients’ previous experiences providing urine samples, knowledge gaps in how to complete a MSCC, and barriers to the clean catch process. Our tools addressed the inconsistencies in instructions and patient gaps in understanding. We also learned our intervention should include assistive devices (eg, upright tray for collection materials, wider collection cups, and a urine collection hat) and/or more intensive clinical staff involvement to address patient physical barriers while completing a clean catch.

As seen in other MSCC studies, instructions prior to urine sample collection are often not provided or are inconsistent and incomplete.^7,8^ When given, instructions are often poorly understood due to low patient literacy and language barriers.^7^ We found similar challenges in patients with deficiencies in anatomical education (ie, inability to identify and subsequently clean the foreskin/labia increasing the potential for contamination during collection). This is concerning as HCPs are often unable to give detailed anatomic description due to time constraints.^2,7^ In a survey done in US emergency rooms, HCPs competing priorities often led to rushed or omitted MSCC instruction.^7^ Our video and flyer were developed to provide detailed description to each patient. Therefore, these may be effective time-saving tools for HCPs in outpatient settings.

Our tools were specifically designed to address issues of literacy and language barriers found in prior studies. Our video and flyers contain pictorial and animated instructions to help overcome literacy barriers, increase comprehension, and improve communication with patients.^20–22^ Illustrations played a pivotal role in bridging the anatomical knowledge gaps in patients by helping them identify genital structures necessary for collecting a midstream sample. Another integral aspect of our tools was the step-by-step guidance providing clear, actionable steps. This appears to have imbued confidence in patients to properly collect a MSCC. Overall, patient input highlighted our tool’s ability to provide clear instructions for a wide demographic of patients.

Patients and providers identified barriers our tools did not address, such as physical inability to complete the instructions. This barrier has been noted in extant literature, however, details on the specific difficult parts of the process have not been explored.^7^ Patients reported difficulty stopping urine flow midstream due to poor stream control, mobility issues due to large body habitus, or older age preventing adequate cleaning of genitalia. Clinic bathroom functionality was also an unexpected barrier that may lead to increased urine culture contamination. Participant solutions to identified barriers included the inclusion of a tray to place collection materials and the addition of a urine hat. While a urine hat is a useful assistive device, it negates the most important part of the clean catch process—the *midstream* collection.^9^ Further research is needed to understand the effects a urine hat has on urine culture contamination.

Our study strengths are multi-method study design and inclusion of a socio-demographically diverse patient population, allowing for a variety of perspectives. Despite a diverse sample, transferability to other clinics may be limited due to the small number of participants included.^23^ Some of the barriers discussed, like available bathroom space for MSCC collection, could vary considerably—even within a given facility. Other limitations of this study included lack of male HCPs’ perspectives on the tools and the fact that we have currently prepared only English and Spanish versions. Next steps for our education tools are to pilot the materials assessing for feasibility, acceptability, and test the effectiveness of our tools for reducing urine contamination in a randomized controlled trial.

## Supporting information

10.1017/ash.2026.10317.sm001 Collazo et al. supplementary material Collazo et al. supplementary material

## Supplementary material

The supplementary material for this article can be found at https://doi.org/10.1017/ash.2026.10317.

## Research transparency and reproducibility

Due to the nature of our qualitative interviews, data will not be made available.
