# Supplementary material for: Patient and healthcare professionals’ perceptions of educational tools to reduce urine culture contamination in outpatient clinics: a qualitative study
Source: Antimicrob Steward Healthc Epidemiol. 2026 Mar 9;6(1):e57. doi: 10.1017/ash.2026.10317 (PMC12975624; doi:10.1017/ash.2026.10317)
Supplement: Collazo et al. supplementary material [file S2732494X26103179sup001.docx]

**Supplemental Material**

*Supplemental Material 1: Midstream Clean Catch Instructional Video Still for Female and Male Patients*

*
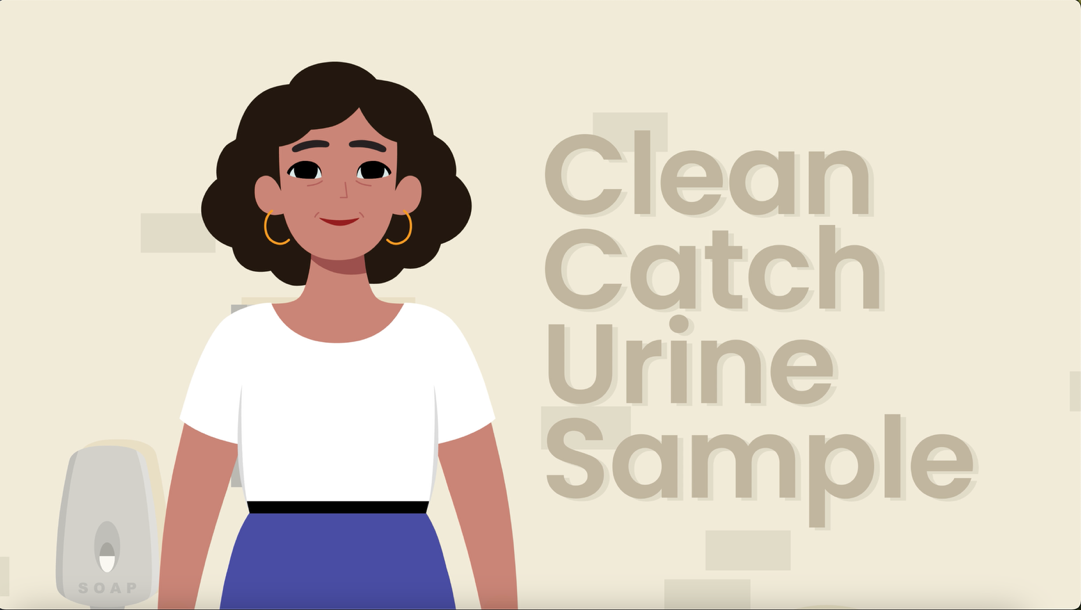
*

*
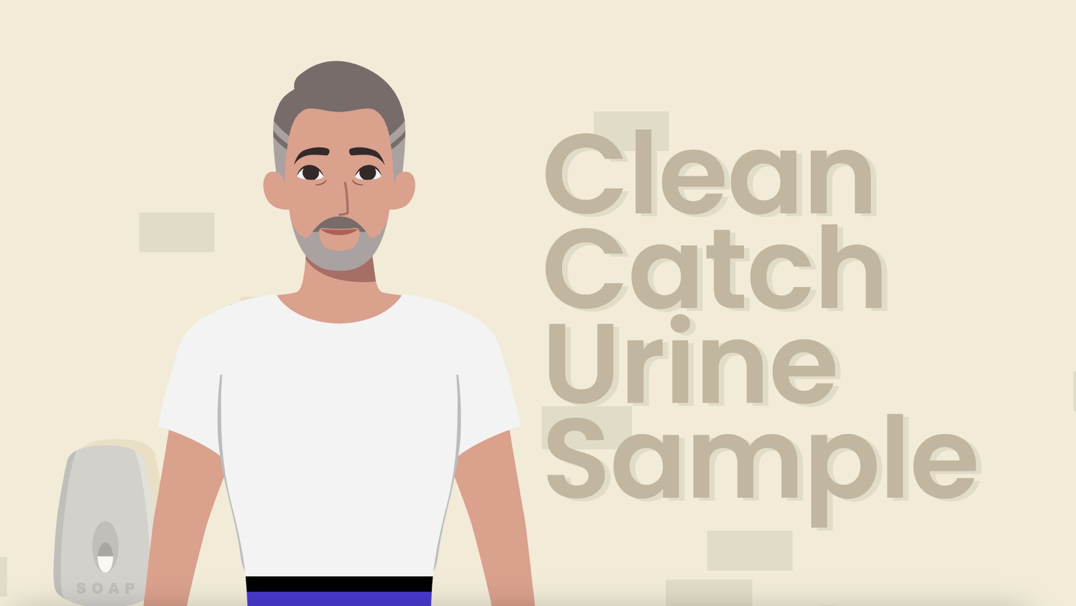
*

*Supplemental Material 2. Interview Guides for Patients and Focus Group Guides for Healthcare Professionals*

PATIENT INTERVIEW GUIDE

**Introduction**

Thank you for taking the time to come in today. We are really interested in learning more about your opinion on our educational video and flyer about how to properly collect a urine sample without contamination using the mid-stream clean-catch method. Just to let you know, this interview is completely voluntary. If there is a question you do not want to answer or if you ever want to stop our discussion, that is completely fine. Just as a reminder, we will be reviewing our materials, which have graphics showing female/male anatomy.

I would also like to record our conversation. Is that okay?

I am going to start the recorder, and then ask you again to make sure we have your response on record.

[If yes, start recorder and ask]: Do you agree to participate in this interview and to have the interview recorded?

Preamble/ Warm up: Thank you again for agreeing to help us with this interview.

Were you able to complete the urine collection using the kit mailed to you at home?

If yes, can you please share your experience using the instructions along with the urine collection kit? Did you have any difficulties with any of the steps?

Probe: If yes, can you please elaborate on this?

Is there anything that you think would be helpful or make the process easier for you or for patients in general?

If not, can you please share the reasons why you were unable to utilize the kit for urine collection?

Probe: Was it due to time or convenience issue? Or unclear instructions on the flyer?

**Research Coordinator - Brief review flyer**

[Sit next to patient go over flyer. Have a pen ready as you review flyer with patient, so that you may circle or mark important questions that they may have. ]

First, distribute the flyer, which was also mailed to the patient, about how to collect a clean urine sample.

**First, show the flyer.**

Distribute the accompanying flyer.

**Think Aloud Interview Questions:**

1. What are your thoughts when you go through the flyer?

**Probe:** Can you tell me what you did or did not like about the flyer?

1. In the flyer, are there any confusing parts or parts that are not clear? Are there any parts that could be clarified or better phrased?
2. Do you have any suggestions for how to improve the flyer?
3. If you were given this flyer during a doctor’s visit, where in the clinic or facility would you want to look at the flyer? [or] Where would you feel comfortable looking at these materials during a doctor’s visit?
4. What are some other things that a clinic could do to make the process of collecting a urine sample easier for patients?

Thank you for that. Now, we’d like to get your input on the video. Play the video:

1. What are your thoughts when you watch the video?

**Probe:** Can you tell me what you did or did not like about the video?

1. In the video, are there any confusing parts or parts that are not clear? Are there any parts that could be clarified or better phrased? (Are any parts of the video that can be simplified?)
2. Do you have any other suggestions for how to improve the video? Can you think of something to enhance the video (or to add to the video) that could help patients collect a clean catch urine sample?
3. If you were asked to view this video during a doctor’s visit, where in the clinic or facility would you want to watch the video? [or] Where would you feel comfortable looking at these materials during a doctor’s visit?
4. Is there anything else you would like to add either about the flyer or video?

HEALTHCARE PROFESSIONL (HCP) FOCUS GROUP GUIDE

I want to welcome everyone to our focus group and thank you all for agreeing to meet with us today and provide your opinions. My name is _____________ and I will be guiding us through this session.

Today we would like to get your insights and suggestions on our educational flyers and videos about how patients can properly collect a urine sample without contamination using the mid-stream clean-catch method. We will ask your opinion on the content of these tools, when and how to distribute these educational materials, and who in your clinic should be involved.

Please note that your participation in this focus group is voluntary. If you do not wish to participate, you may leave at this time. Your decision of whether or not to participate will not affect your employment, performance evaluation, or any other employment practice.

Before we start, we’d like to ask each of you for your permission to record our discussion. We will be recording full facial features and voice. You may receive personal and professional satisfaction through contributing to health research. However, you may receive no direct benefit from your participation in this study. The main risk associated with this study includes loss of confidentiality since the discussion will take place in a group setting. However, the study personnel will make every effort to minimize these risks. We ask that you refrain from using any names during this session to maintain anonymity. We will also ask that you respect the privacy of other participants and not repeat what is said during this focus group. Researchers will remove any identifiable information before transmitting any research data to sponsors. PHI will not be disclosed or shared with any other person or entity, except as required by law, for authorized oversight of the research study, or for other research for which the use of disclosure of the PHI would be permitted under the Privacy Rule. All patient interview recordings and transcripts as well as focus group recordings and transcripts will be shared only with the research team using a password-protected shared OneDrive folder on the secure Baylor network. Audio recordings will be destroyed within a year of completed data analysis. The recordings collected for this project will be used for the qualitative research analysis. This analysis is part of our project aimed at developing a patient educational video and flyer to give step-by-step instructions for mid-stream clean catch urine collection. Recordings will not be used for educational or commercial purposes. We will then show the flyers and videos. Please keep in mind that these materials have anatomical graphics for males and females. Please give us a thumbs up, either on screen or through your emoji.

If you have any questions about this study, please contact Dr. Larissa Grigoryan at (713) 798-3293. If you have additional questions about your rights as a research participant, contact the Institutional Review Board for Human Subject Research for Baylor College of Medicine & Affiliated Hospitals at (713) 798-6970 or [irb@bcm.edu](mailto:irb@bcm.edu).

Thank you, let’s begin.

We have sent you the draft flyers and videos that we plan to distribute at Harris Health and Baylor clinics. These materials will be given to some patients who need to provide a clean-catch urine sample. We will begin with your feedback on the flyers.

Flyers Questions:

1. **Can you tell me what you did or did not like about the flyers?**
2. What do you think about the flyers’ layout/color/graphics?
3. In the flyers, are there any confusing parts or sections that are not clear?
4. How would you make these more clear? Any suggestions on wording?
5. **Would you use these flyers in clinic with patients? Why or why not?**

[If they respond positively]:

1. Can you tell me where and how you might use these flyers?
2. How would these flyers fit into your existing workflow?

[If they respond negatively]:

1. Can you tell us why you would not use these flyers? (probes—lack of time, distasteful, not needed)
2. **When and who do you think would be best to review the flyer with patients?**
3. Could patients review the flyers on their own?
4. What verbal instructions do you think clinic personnel should offer as they show these flyers to patients?
5. Is there anything else that you would like to add or let us know about how we can improve these flyers?

**Play video – warning for graphic images (Female video first, then comments; followed by the male video + comments) (we can alternate the order for multiple focus groups)**

Video Questions

1. **Can you tell me what you did or did not like about the videos?**
2. What do you think about the videos’ layout/color/graphics?
3. In the videos, are there any confusing parts or parts that are not clear?
   1. How would you make these more clear? Any suggestions on wording?
4. **Would you use these videos in clinic with patients? Why or why not?**

[If they respond positively]:

1. Can you tell me where and how you might use these videos?
2. How would these videos fit in your existing workflow?

[If they respond negatively]:

1. Can you tell us why you would not use these videos? (probes—lack of time, distasteful, not needed)
2. **When and who do you think would be best to review the video with patients?**
3. Could patients review the video on their own?
4. What verbal instructions do you think clinic personnel should offer as they show these videos to patients?
5. Is there anything else that you would like to add or let us know about how we can improve these videos?

**Implementation Questions about the flyers and videos:**

**Preface:** Your feedback will inform the future of this project. Our research team will be provide all resources needed for the trial, which we will carry out.

**For the video, the coordinator will operate the tablet for the patient to view it.**

1. **For the flyer, if you think clinic personnel should not be involved in distributing it, what steps would you recommend making sure this gets into the hands of patients at your clinics?**
2. **Do you think these materials will be useful in clinic with patients?**
3. **What do you think we could do to make the urine culture collection process easier for patients?**

**Finally:** We are hoping to pilot distribution of these flyers and videos at two Harris Health clinics (or Baylor Family Medicine clinics).

1. **How can we encourage staff to participate in our pilot program? What might be some useful benefits or incentives for participation?**
